# Expression of *Arabidopsis FCS-Like Zinc finger* genes is differentially regulated by sugars, cellular energy level, and abiotic stress

**DOI:** 10.3389/fpls.2015.00746

**Published:** 2015-09-24

**Authors:** Muhammed Jamsheer K, Ashverya Laxmi

**Affiliations:** National Institute of Plant Genome Research, New DelhiIndia

**Keywords:** *Arabidopsis*, *FLZ* gene family, sugar signaling, energy signaling, SnRK1, low-energy stress, abiotic stress, *HXK1*

## Abstract

Cellular energy status is an important regulator of plant growth, development, and stress mitigation. Environmental stresses ultimately lead to energy deficit in the cell which activates the SNF1-RELATED KINASE 1 (SnRK1) signaling cascade which eventually triggering a massive reprogramming of transcription to enable the plant to survive under low-energy conditions. The role of *Arabidopsis thaliana FCS-Like Zinc finger* (*FLZ*) gene family in energy and stress signaling is recently come to highlight after their interaction with kinase subunits of SnRK1 were identified. In a detailed expression analysis in different sugars, energy starvation, and replenishment series, we identified that the expression of most of the *FLZ* genes is differentially modulated by cellular energy level. It was found that *FLZ* gene family contains genes which are both positively and negatively regulated by energy deficit as well as energy-rich conditions. Genetic and pharmacological studies identified the role of *HEXOKINASE 1*- dependent and energy signaling pathways in the sugar-induced expression of *FLZ* genes. Further, these genes were also found to be highly responsive to different stresses as well as abscisic acid. In over-expression of kinase subunit of *SnRK1*, *FLZ* genes were found to be differentially regulated in accordance with their response toward energy fluctuation suggesting that these genes may work downstream to the established *SnRK1* signaling under low-energy stress. Taken together, the present study provides a conceptual framework for further studies related to *SnRK1-FLZ* interaction in relation to sugar and energy signaling and stress response.

## Introduction

Life is modulated by an array of internal and external factors which act as signals to control the fate of the organisms. In response to these factors, organisms tone their growth and reproduction so that they will effectively survive and reproduce. Cellular energy status is directly dependent on the supply of sugars. Internal sugar status is an important factor which controls the lifespan and aging in metazoans, yeast and plants (Heilbronn and Ravussin, 2005; Minina et al., 2013). Adequate sugar supply is required for the better stress tolerance. Glucose regulates a large number of genes involved in both biotic and abiotic stress response (Price et al., 2004). Sucrose promotes the survival of plants under salt stress through G-protein signaling (Colaneri et al., 2014). Many of the mutants defective in sugar responses were found to be allelic to mutants of abscisic acid (ABA) and ethylene signaling suggesting a large interplay between sugar and stress signaling (Leon and Sheen, 2003).

In plants, stressful environment ultimately leads to energy deprivation in the cell which activates the evolutionarily conserved multi-subunit serine-threonine kinase SNF1-RELATED KINASE 1 (SnRK1). SnRK1 acts a central integrator of energy metabolism, stress, and developmental cues (Baena-González et al., 2007). Similar to its mammalian homolog AMP-activated protein kinase (AMPK), SnRK1 was also found to be phosphorylated on its T-loop and its dephosphorylation is inhibited by 5′-AMP (Sugden et al., 1999; Hey et al., 2007). In response to low-energy stress, SnRK1 promotes catabolism and minimize anabolism through reprogramming the transcriptional machinery which enables the plants to survive under low-energy conditions (Baena-González et al., 2007). It was also found that SnRK1 imparts tolerance to various biotic as well as abiotic stresses via controlling the transcription of genes involved in stress tolerance (Baena-González et al., 2007; Baena-González and Sheen, 2008; Cho et al., 2012; Confraria et al., 2013). SnRK1 activity is essential for establishing flooding stress tolerance in rice (Cho et al., 2012). A PP2C hub is involved in the regulation of both SnRK1 and ABA signaling suggesting an integrated molecular pathway in the mitigation of low-energy condition and abiotic stresses (Rodrigues et al., 2013).

The plant-specific FCS-like zinc finger (FLZ) domain proteins are implicated in the regulation of various biotic and abiotic stresses. *FCS-LIKE ZINC FINGER 9/MEDIATOR OF ABA-REGULATED DORMANCY 1* (*FLZ9/MARD1*) is found to be involved in the ABA-mediated seed dormancy and up-regulated during leaf senescence (He et al., 2001; He and Gan, 2004). Over-expression of *FCS-LIKE ZINC FINGER 4/INCREASED RESISTANCE TO MYZUS PERSICAE 1* (*FLZ4/IRM1*) rendered plants shorter which creates mechanical resistance to aphid attack (Chen et al., 2013). Over-expression of a wheat *FLZ* gene, salt-related hypothetical protein (*TaSRHP*) in *Arabidopsis thaliana* resulted in enhanced resistance to salt and drought stress (Hou et al., 2013). The expression analysis from the publically available microarray data suggests that members of *A. thaliana FLZ* gene family is responsive to ABA, JA, various abiotic stresses, glucose, and nitrogen and phosphorous deficiency (Nietzsche et al., 2014). Protein–protein interaction studies identified that all 18 *A. thaliana* FLZ proteins interact with kinase subunits of SnRK1 (Arabidopsis Interactome Mapping Consortium, 2011; Nietzsche et al., 2014).

The available evidence indicates the possible role of *FLZ* gene family in stress tolerance and adaptive growth. Similarly, their physical interaction with kinase subunits of SnRK1 suggests their relation with SnRK1 signaling. However, their relation with SnRK1 signaling particularly during energy fluctuations in the cell and abiotic stresses is not explored yet. In this study, we analyzed the transcriptional regulation of *FLZ* gene family during low-energy stress and energy rich conditions. We also identified that different sugar signaling pathways regulate the sugar-dependent transcription of *FLZ* genes. We also analyzed the expression of these genes under ABA treatment and different stresses with particular emphasize on salt stress. Over-expression of kinase subunit of SnRK1 resulted in differential regulation of some of the *FLZ* genes suggesting that SnRK1 signaling transcriptionally regulates these genes in the plants.

## Materials and Methods

### Plant Material and Growth Conditions

The *A. thaliana* Columbia (Col-0) and Landsberg erecta (Ler) ecotypes were used as controls in the experiments. All the experiments were done in Col-0 unless stated. Seeds of *gin2-1* (CS6383) were obtained from ABRC (https://abrc.osu.edu; Moore et al., 2003). *KIN10 OE2* seeds were provided by Prof. Filip Rolland (Metabolic signaling group, KU Leuven, Baena-González et al., 2007). For all experiments, seeds were surface sterilized and stored at 4°C for 48 h in dark for stratification. The imbibed seeds were grown on square petri plates containing 0.5X MS medium with 1% sucrose and 0.8% agar. The plates were kept vertically for germination and growth in climate-controlled growth room under 16:8 h photoperiod with 22 ± 2°C temperatures and 60 μmol m^-2^ s^-1^ light intensity unless stated. Five-days old seedlings grown in standard growth conditions were used for all experiments and at least 40 seedlings were harvested for each sample. *KIN1OE2* and Ler seedlings grown for 5 days in the standard growth conditions were used for gene expression analysis.

### Sugar Starvation and Replenishment Assay

Five-days old uniformly grown Col-0 seedlings under standard growth condition were used for sugar starvation and replenishment assay. Plants were starved in 0.5X MS liquid medium without sucrose in 22°C at 140 rpm in darkness. Samples were collected after 3, 6, 12, and 24 h time points of starvation. After 24 h time point, the plants were transferred to 0.5X liquid medium with sucrose and grown under 22°C at 140 rpm in the light. Samples were collected after 3, 6, 12, and 24 h time points of replenishment.

### Sugar Sensitivity Assays and Treatment with Chemical Inhibitors

The sugar sensitivity assay is done as described previously using 3% glucose/sucrose/3-*O*-methylglucose/mannose/mannitol individually (Mishra et al., 2009). For sugar sensitivity assay in glucose signaling mutant *gin2-1*, the Ler and *gin2-1* seedlings were subjected to the same treatment. For sugar sensitivity assay along with metabolic inhibitors, Col-0 seedling were transferred to 0.5X MS medium with 3% glucose or sucrose with 3% 2-Deoxy-D-glucose (2DG)/5 μM Antimycin-A (AmA)/50 μM 2,4-Dinitrophenol (DNP)/10 μM Carbonyl cyanide *m*-chlorophenyl hydrazone (CCCP) (Sigma–Aldrich) individually in the same treatment condition mentioned above. For 2DG treatment, 5-days old Col-0 seedlings grown in standard growth conditions were treated with 25, 50, and 100 mM 2DG in 0.5X liquid MS medium in the dark for 3 h at 140 rpm.

### Abscisic Acid and Salt Treatments

Five-days old uniformly grown Col-0 seedlings grown under standard growth condition were used for ABA as well as NaCl treatment. The seedlings were treated with 10 μM ABA in 0.5X liquid MS medium for 30 min, 3 and 6 h time points at 22°C at 140 rpm in the light. For salt treatment, the seedlings were subjected to 150 mM NaCl treatment in 0.5X liquid MS medium for 3 h at 22°C at 140 rpm in the light.

### Gene Expression Analysis

RNA isolation and cDNA preparation were performed as described previously (Mishra et al., 2009). qRT-PCR were done with 1:50 diluted cDNA samples with SYBR-Green PCR master mix in 384-well optical reaction plates employing Applied Biosystems 7500 Fast Real-Time PCR System (Applied Biosystems, USA). The primers were prepared from the transcript sequence of genes using PRIMER EXPRESS version v3.0 (Applied Biosystems, USA) with default parameters. *18S rRNA* and *UBQ10* were as used endogenous controls and relative quantification of the mRNA level of candidate genes were calculated by ΔΔCT method (Livak and Schmittgen, 2001). Primers used for qRT-PCR experiments are given in Supplementary Table S1. The heat maps were generated from the gene expression data using MultiExperiment Viewer (MeV, v4.8; Saeed et al., 2006). The hierarchical clustering of genes was performed by Pearson correlation algorithm with average linkage clustering.

The digital gene expression analysis under ABA and abiotic stress treatments was done using the AtGenExpress data available through *Arabidopsis* eFP Browser (Kilian et al., 2007; Winter et al., 2007; Goda et al., 2008). The expressions in the treatment time points were calculated using control expression values and heat map and hierarchical clustering were done as described above.

### Promoter: *GUS* Line Construction and Starvation Assay

For promoter: *GUS* transcriptional fusion constructs (p*FLZ1*::*GUSA*, p*FLZ6*::*GUSA*, and p*FLZ8*::*GUSA*), primers designed from 2 kb 5′ UTR upstream region of *FLZ1* and *FLZ6* and 1.5 kb 5′ UTR upstream region of *FLZ8*. Primers used for cloning are listed in Supplementary Table S1. The amplified products were cloned in pMDC164 vector and transformed into Col-0 plants through floral dip transformation method (Clough and Bent, 1998; Curtis and Grossniklaus, 2003). The transformants were selected on 0.5X MS plates supplemented with 15 μg/ml hygromycin A and homozygous lines were identified in T3 generation. For starvation assay of promoter:: GUS lines, leaves of the same developmental stage from the rosette plants grown in standard growth conditions were detached and kept in petri plates with soaked filter paper in the dark for 1 and 2 days. The leaves were GUS stained for 1 h as described previously (Jefferson et al., 1987).

## Results

### Response of *Arabidopsis thaliana FLZ* Gene Family Genes Toward Cellular Energy Fluctuation

**FIGURE 1:**
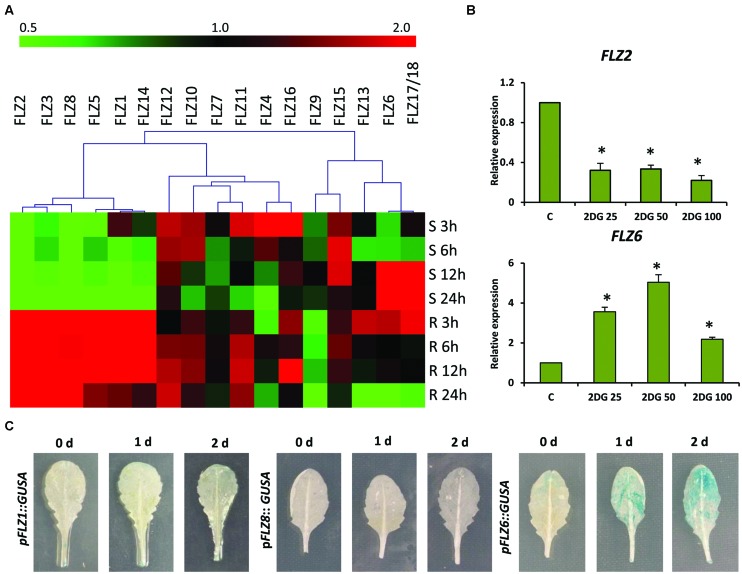
**Expression of *Arabidopsis thaliana FLZ* gene family during low-energy stress and replenishment. (A)** Expression of *A. thaliana FLZ* gene family under energy starvation and replenishment studied with qRT-PCR. S denotes ‘starvation’ and R denotes ‘recovery.’ The expression in 0.5X MS grown plants was taken as the control to compare the expression in low-energy levels and replenishment series. The genes were subjected to hierarchical clustering based on their response toward these treatments. *UBQ10* was used as endogenous control. **(B)** Expression of *FLZ2* and *FLZ6* in response to increasing concentration of 2DG (25, 50, and 100 mM). The bars represent the average of two independent biological experiments with three technical replicates each and error bars represent SE. *UBQ10* was used as endogenous control. Asterisk indicates a significant difference of expression in the treatment compared to control (*P* < 0.005, Student’s *t*-test). **(C)** The response of *pFLZ1*::*GUSA*, *pFLZ8*::*GUSA*, and *pFLZ6*::*GUSA* transcriptional-reporter lines toward energy starvation for 0, 1, and 2 days.

The microarray-based expression analysis and protein–protein interaction analysis with KIN10/11 suggests the involvement of *FLZ* genes in low-energy stress (Nietzsche et al., 2014). In order to study the response of *FLZ* gene family genes in low-energy stress at the transcriptional level, we did an extensive energy depletion and replenishment assay using 5-days old Col-0 seedlings (Supplementary Figure S1A). The expression of all 18 *A. thaliana FLZ* genes was analyzed at different time points of low-energy stress and energy replenishment conditions (**Figure 1A**). It was found that the transcript levels of a group of genes were rapidly decreased on the onset of low-energy stress and these genes formed a distinct cluster in the heat map. The expression of these genes was significantly down-regulated in response to mild as well as prolonged energy depletion and their expressions were rapidly up-regulated in response to sugar replenishment (Supplementary Figure S1B). *FLZ1, FLZ2, FLZ3, FLZ5, FLZ8*, and *FLZ14* belong to this cluster of genes whose levels are rapidly perturbed in response to energy depletion and replenishment. Among other members of this gene family, *FLZ12*, *FLZ7*, and *FLZ15* do not show any significant difference in the expression in all treatments studied. *FLZ4*, *FLZ10*, *FLZ11*, and *FLZ16* showed significant up-regulation of transcript level in the mild energy depletion. However, prolonged energy depletion significantly reduced their expression and sugar replenishment restored their expression to control level in most of these genes. *FLZ9* showed a mild repression of transcript level at the onset of energy depletion; however, it showed significant repression of transcript levels after sugar replenishment. Interestingly, prolonged starvation significantly induced the transcript levels of *FLZ6* and *FLZ17*/18 suggesting that these genes may be involved in the regulation of growth under prolonged low-energy stress. Consistent with this, it was found that the level of these two genes gradually reached the control level after energy is replenished to the plant. The levels were further reduced during prolonged incubation in energy-rich conditions (**Figure 1A**).

In order to validate the results obtained from energy depletion and replenishment assay, the expression of two selected *FLZ* genes was analyzed in samples treated with increasing concentrations of glycolysis inhibitor 2DG (**Figure 1B**). 2DG is a non-metabolizable glucose analog which is also a substrate of hexokinase and blocks the glycolytic pathway through competitive inhibition which ultimately leads to energy depletion in the cell (Wick et al., 1957). Increasing concentrations of 2DG significantly repressed the level of *FLZ2* which was found to be negatively regulated during low-energy stress in the previous experiment. *FLZ6*, which was found to be positively regulated by prolonged low-energy stress, was found to be significantly up-regulated in response to 2DG treatment. These results validate the observations of the previous experiment. In order to further confirm these results, we used reporter lines of starvation-repressible and sugar-inducible genes *FLZ1* and *FLZ8 (pFLZ1::GUSA* and *pFLZ8*::*GUSA*) and prolonged starvation-inducible gene *FLZ6* (p*FLZ6*::*GUSA*). Uniformly grown leaves of the same developmental stage were subjected to low-energy stress for 1 and 2 days and the GUS activity was compared with the leaves grown in normal conditions (**Figure 1C**). The GUS activity was found to be too low in the untreated leaves which is consistent with the earlier observation that the expression of these genes are too low in the mature leaves compared to other tissues (Jamsheer et al., 2015). The starvation could not induce the GUS activity in the leaves of *pFLZ1::GUSA* and p*FLZ8*::*GUSA* lines. However; low-energy stress induced GUS expression in p*FLZ6*::*GUSA* line. All these results conclusively suggest that *FLZ* gene family contain genes which are positively and negatively regulated during low and high energy levels in the plant.

### Response of *FLZ* Genes Toward Sugars

In the energy depletion and replenishment assay, it was found that the expression of many genes was positively or negatively regulated by sucrose replenishment. In order to dissect how the *FLZ* genes respond to sugar and energy rich condition in the cell, 24 h energy-starved Col-0 plants were treated with glucose, sucrose, mannose, and sugar alcohol mannitol. In order to rule out the effects of sugars other than the nutrient effect, seedlings were also treated with a non-metabolizable and non-toxic glucose analog, 3-*O*-methyl glucose (3-OMG; Cortès et al., 2003). Further, mannitol was used as an additional osmotic control to rule out the involvement of osmotic regulation of *FLZ* genes in the results. Among sugars, glucose, and sucrose profusely altered the transcript level of many genes (Supplementary Figure S2).

**FIGURE 2:**
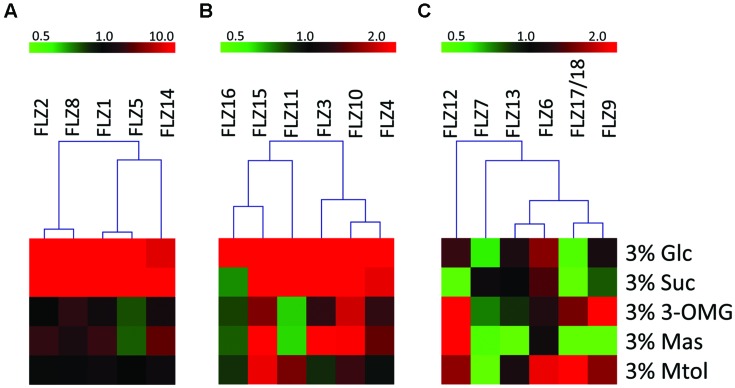
**Sugar sensitivity of *Arabidopsis thaliana FLZ* gene family.** The *A. thaliana FLZ* gene family was classified into three groups based on their response toward sugar treatments in qRT-PCR experiments. The expression in 0% sugar grown plants was taken as the control for comparing the expression in other treatments and *18S rRNA* was used as endogenous control. **(A)** High sugar-inducible genes (Class I, induced by ≥10 fold by glucose and/or sucrose). **(B)** Medium sugar-inducible genes (Class II, induced by ≥2 fold by glucose and/or sucrose). **(C)** Sugar non-responsive and sugar-repressible genes (Class III, significantly repressed by glucose and/or sucrose).

Based on their response toward glucose and/or sucrose treatment, the *FLZ* gene family was divided into three distinct classes. The transcript level of class I genes was induced by ≥10 fold by glucose and/or sucrose (**Figure 2A**). *FLZ1, FLZ2, FLZ5, FLZ8*, and *FLZ14* genes belong to this class of high sugar-inducible genes. Class II genes are medium sugar-inducible genes which are induced by ≥2 fold by glucose and/or sucrose (**Figure 2B**). Class III includes sugar non-responsive genes as well as sugar-repressible genes (**Figure 2C**). *FLZ6* could not display any significant response toward sugar treatments (Supplementary Figure S2). Interestingly, the levels *FLZ7*, *FLZ9*, *FLZ12*, and *FLZ17*/18 were found to be significantly decreased in glucose, sucrose or in both sugars suggesting that these genes are sugar-repressed genes.

Mannose could also affect the transcription of many genes albeit to a lesser extent (Supplementary Figure S2). Among the sugar-induced genes, *FLZ1*, *FLZ2*, *FLZ3*, *FLZ4* were found to be up-regulated in the presence of mannose. Similarly, sugar down-regulated genes such as *FLZ7*, *FLLZ9*, and *FLZ17*/18 were found to be also downregulated by mannose. *FLZ12*, which is specifically repressed by sucrose showed highest positive response toward mannose treatment.

### Role of Different Sugar Signaling Pathways in the Sugar Responsiveness of *FLZ* Genes

It is already identified that different sugar signaling pathways act individually or along with other sugar signaling pathways to regulate the expression of sugar-responsive genes. The role of HXK1-dependent glucose signaling pathway is attributed to many glucose-mediated responses in plants (Mishra et al., 2009). Consistent with this, the glucose-induced up-regulation of key transcription factors involved in the aliphatic glucosinolate biosynthesis is completely abolished in the *gin2-1* mutant (Miao et al., 2013). Similarly, the glucose-dependent repression of *TANDEM ZINC FINGER 1* transcription is dependent on HXK1-signaling (Lin et al., 2011). These reports suggest that downstream effects of glucose at the transcriptional level are partly mediated by HXK1-dependent glucose signaling pathway. Similarly, the role of TOR and SnRK1 dependent energy signaling pathway is also implicated in the regulation of many sugar responsive genes (Baena-González et al., 2007; Ramon et al., 2008; Xiong et al., 2013).

**FIGURE 3:**
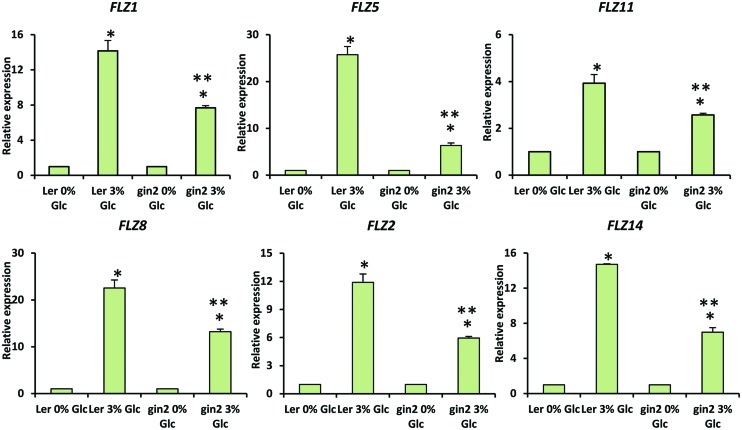
**Involvement of HXK1-dependent glucose signaling in the regulation of glucose-dependent up-regulation of *FLZ* genes.** The effect of *gin2-1* mutation on the expression of glucose-responsive *FLZ* genes was studied with qRT-PCR. The expression in 0% sugar treatment in Ler and *gin2-1* were taken as the control individually for comparing the expression in 3% glucose treatment in WT and mutant respectively. *18S rRNA* was used as endogenous control. The bars represent the average of two independent biological experiments with three technical replicates each and error bars represent SE. Single asterisk indicates a significant difference of expression in the treatment compared to control 0% sugar. Double asterisk indicates a significant difference of expression in the *gin2-1* mutant after the treatment compared to the treatment in Ler (*P* < 0.005, Student’s *t*-test).

**FIGURE 4:**
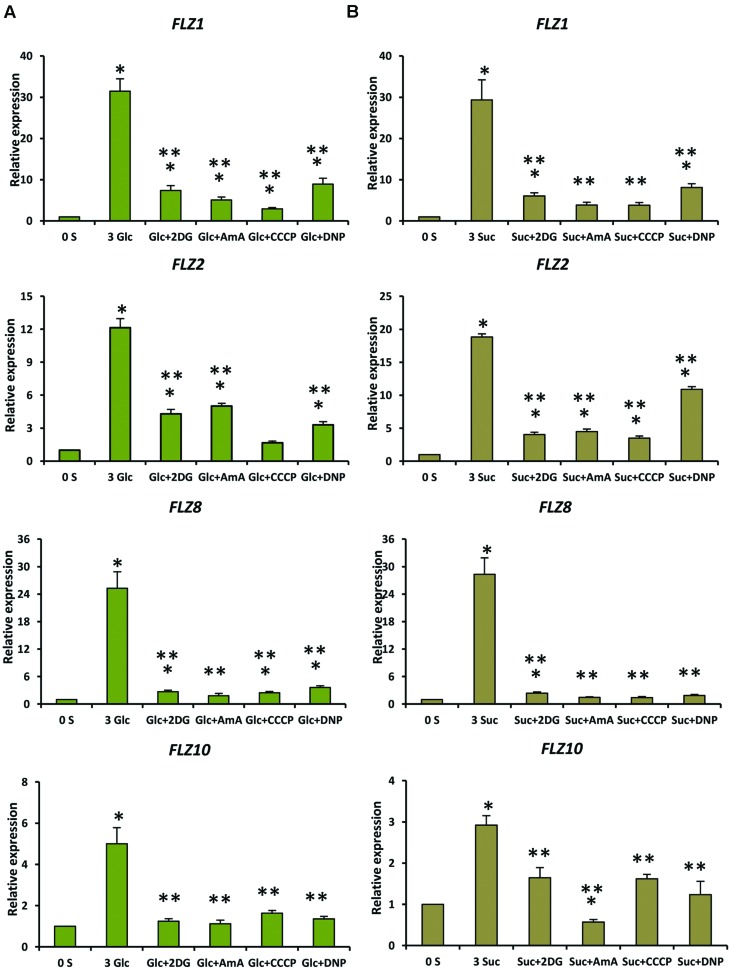
**Involvement of energy signaling in the regulation sugar-dependent up-regulation of *FLZ* genes.** The effect of metabolic inhibitors on the sugar-mediated transcriptional up-regulation of *FLZ* genes was studied by qRT-PCR. **(A)** The effect of metabolic inhibitors on the glucose-sensitivity of *FLZ* genes. **(B)** The effect of metabolic inhibitors on the sucrose-sensitivity of *FLZ* genes. The expression in 0% sugar grown plants was taken as the control for comparing the expression in 3% sugar treatment alone or with metabolic inhibitors. *UBQ10* was used as endogenous control. The bars represent the average of two independent biological experiments with three technical replicates each and error bars represent SE. Single asterisk indicates a significant difference of expression in the treatment compared to control 0% sugar. Double asterisk indicates a significant difference of expression in the treatment compared to 3% Glc or 3% Suc (*P* < 0.005, Student’s *t*-test).

Genetic and pharmacological approaches were used to identify the role of different sugar signaling pathways on the sugar responsiveness of *FLZ* genes. To study whether HXK1-dependent glucose signaling has any role in the transcriptional up-regulation of *FLZ* genes, we checked the glucose responsiveness of sugar-inducible *FLZ* genes in the *gin2-1* mutant. It was found that the transcript induction of *FLZ* genes after glucose treatment was significantly reduced in the *gin2-1* mutant (**Figure 3**). Abolition of the HXK1-dependent glucose signaling pathway could only partially affect the sugar-dependent activation of *FLZ* genes suggesting the involvement of other sugar signaling pathways in this response. The treatment of 2DG significantly reduced the transcript level of *FLZ2* which suggest that ultimately cellular energy level regulates the expression of sugar-induced *FLZ* genes. To confirm this observation, sugar depleted seedlings were treated with sugars alone or in combination with chemicals which inhibit glycolysis and various steps of oxidative phosphorylation. The glycolysis inhibitor 2DG, the mitochondrial electron transport blocker AMA and mitochondrial uncoupling agents CCCP and DNP were used for studying the effect of metabolic signaling on the induction of *FLZ* genes (Xiong et al., 2013). It was found that the induction of transcript level by sugars was severely abolished when sugars were given in combination with metabolic inhibitors (**Figure 4**).

### Response of *FLZ* Genes towards Different Abiotic Stresses and ABA Treatment

**FIGURE 5:**
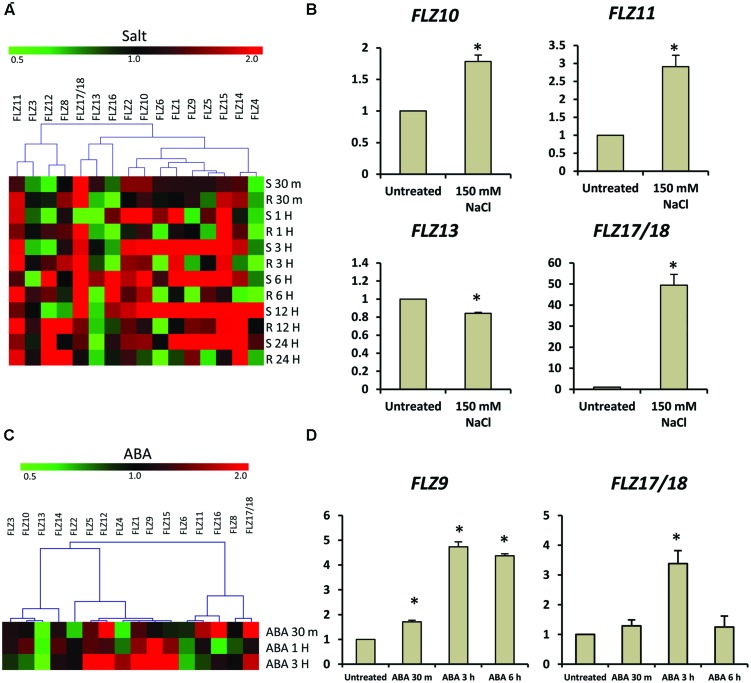
**Expression of *FLZ* genes during salt stress and abscisic acid.** The impact of salt stress and ABA on the expression of *FLZ* genes was studied using available microarray data and by qRT-PCR. **(A)** The expression of *FLZ* genes during salt stress from available microarray data. The expression data was downloaded from the AtGenExpress global stress expression data set (Kilian et al., 2007) available through *Arabidopsis* eFP Browser (Winter et al., 2007). The genes were subjected to hierarchical clustering based on their response toward salt stress. S denotes ‘shoot’ and R denotes ‘root.’ **(B)** The expression of selected *FLZ* genes in 5-days old seedlings under salt stress. **(C)** The response of *FLZ* genes toward ABA from available microarray data available through *Arabidopsis* eFP Browser (Winter et al., 2007; Goda et al., 2008). **(D)** The expression of two selected *FLZ* genes in 5-days old seedlings treated with ABA at three different time points. In both salt stress and ABA treatments, the expression in 5-days old Col seedlings grown on 0.5X MS was used as the control to compare the transcript level of *FLZ* genes under salt stress and ABA. *18S rRNA* was used as endogenous control. The bars represent the average of two independent biological experiments with three technical replicates each and error bars represent SE. Asterisk indicates a significant difference of expression in the treatment compared to control (*P* < 0.005, Student’s *t*-test).

Over-expression of a wheat *FLZ* gene *TaSRHP* in *A. thaliana* resulted in enhanced salt stress tolerance. *TaSRHP* found to be a positive regulator of many stress-related genes (Hou et al., 2013). This result suggests an involvement of *FLZ* genes in salt stress response. Most of the *FLZ* genes were found to be responsive to the cellular energy deficit which is also caused by stresses. We made use of available microarray data to investigate whether salt stress directly regulates the expression of *FLZ* genes and identified that salt stress differentially regulate the expression of all *FLZ* genes (**Figure 5A**). Genes like *FLZ2*, *FLZ10*, *FLZ11*, and *FLZ15* were found to be constantly up-regulated during salt stress. However, most of the genes showed temporal response toward the salt treatment. In order to validate the microarray data, 5-days old seedlings were subjected to salt stress for 3 h and the expression level of 4 *FLZ* genes which were shown differential regulation in the microarray data was analyzed (**Figure 5B**). As observed in the microarray data, *FLZ10* and *FLZ11* were found to be moderately up-regulated in response to 3 h salt stress. Similarly, moderate down-regulation of *FLZ13* was observed in the qRT-PCR data also. *FLZ17*/*18*, which were found to be profusely up-regulated in almost all stages in the microarray data was found to be profusely up-regulated in the salt treated 5-days old seedlings too.

We also analyzed the expression pattern of *FLZ* genes under other abiotic stress using available microarray data and it was found that the expression of these genes were considerably fluctuated in response to different abiotic stresses (Supplementary Figure S3). In general, it can be seen that the response of these genes toward different stresses are more or less spatiotemporal. The varied response of *FLZ* genes toward different abiotic stress prompted us to investigate the response of these genes toward ABA. Digital expression analysis identified that many members are of this gene family are differentially regulated in response to ABA treatment (**Figure 5C**). Most of the genes were found to be positively regulated by ABA while few genes like *FLZ13* were found to be down-regulated. In order to validate the microarray data, we analyzed the response of two *FLZ* genes in 5-days old seedlings treated with ABA at three different time points (**Figure 5D**). Among these two genes, the ABA-responsiveness of *FLZ9* is already reported earlier which can be used as excellent marker for validating microarray data (He and Gan, 2004). As observed in the microarray data, the expression of *FLZ9* was found to be gradually increased in response to ABA treatment. Similarly, the expression of *FLZ17/18* was also found to be significantly increased in response to 3 h ABA treatment. Taken together, all these results suggest the possible involvement on *FLZ* genes in plant growth under stress.

### Involvement of SnRK1 in the Regulation of *FLZ* Genes

**FIGURE 6:**
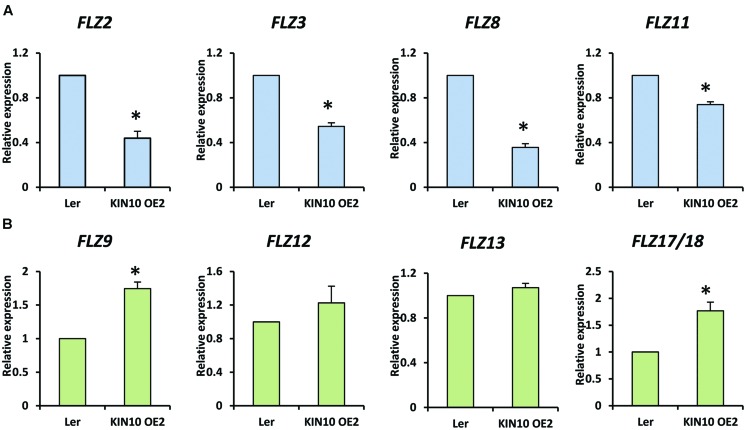
**Expression of *FLZ* genes in SnRK1.1 over-expression line.** The transcript level of selected *FLZ* genes in *KIN10 OE2* seedlings were studied with qRT-PCR. **(A)** The expression of sugar-inducible (Class I and II) *FLZ* genes in *KIN10 OE2*. **(B)** The expression of Class III *FLZ* genes in *KIN10 OE2*. The expression in 5-days old Ler was used as the control to compare the transcript level of *FLZ* genes in *KIN10 OE2*. *UBQ10* was used as endogenous control. The bars represent the average of two independent biological experiments with three technical replicates each and error bars represent SE. Single asterisk indicates a significant difference of expression in *KIN10 OE2* compared to Ler (*P* < 0.005, Student’s *t*-test).

It is already established that SnRK1 regulates the expression of genes involved in the mitigation of multiple stresses including low-energy stress (Baena-González et al., 2007). The over-expression of SnRK1.1 in *A. thaliana* resulted in enhanced starvation tolerance, late senescence, and perturbation in the normal developmental processes (Baena-González et al., 2007). In order to investigate whether the *FLZ* genes have any connection with the conserved SnRK1 signaling cascade, we checked the expression of selected *FLZ* genes from all three classes in *KIN10* over-expression line *KIN10 OE2* (Supplementary Figure S4). It was found that SnRK1 repress the transcription of sugar-inducible *FLZ* genes (**Figure 6A**). The degree of repression in high sugar-inducible genes (Class I) like *FLZ2*, *FLZ3*, and *FLZ8* were found to be more severe compared to the repression in medium sugar-inducible gene (Class II) *FLZ11*. The expression of sugar-repressible genes (Class III) *FLZ9* and *FLZ17*/*18* were found to be significantly increased in *KIN10* over-expression line (**Figure 6B**). However, the expression of sucrose-repressible and mannose-inducible gene *FLZ12* was found to be unperturbed. Mannose-repressible *FLZ13* level was also found to be unchanged in the *KIN10* over-expression lines suggesting that the regulation of SnRK1 on *FLZ* gene is directly dependent on their response toward metabolizable sugars such as glucose and sucrose.

## Discussion

### *FLZ* Genes are Transcriptionally Regulated by Sugars, ABA, and Abiotic Stresses

It is already reported that *FLZ9* expression is induced during leaf senescence and nutrient deficiency differentially regulates *FLZ* genes (He et al., 2001; Nietzsche et al., 2014). In our analysis, it was found that the expression of most of the *FLZ* gene family members is differentially regulated in response to sugar treatment and under energy depleted and replenished conditions. The expressions of these genes were found to be highly regulated by glucose, sucrose and to a very less extent by mannose which confirms that this response is dependent on sugar signaling and metabolism but not dependent on the osmotic effects of sugars. We could classify the *FLZ* genes into three classes based on their response toward sugars. Each class contains genes in which some of them is phylogenetically closer and some of them are phylogenetically very distant suggesting that the specific response toward sugars is not confined to any specific clad defined in an earlier study (Jamsheer and Laxmi, 2014). The expression of all high sugar-inducible genes (Class I) was repressed in response to low-energy stress and their levels were rapidly increased during sugar replenishment. Similarly, *FLZ3* which is a paralog of high sugar-inducible genes *FLZ1* and *FLZ2* showed a similar response. In medium sugar-inducible group (Class II), many genes found to be were up-regulated in response to mild low-energy stress while prolonged stress repressed their levels. Some of the sugar-repressible genes (Class III) identified from the sugar sensitivity assay showed induction of transcript levels in response to low-energy stress. *FLZ6* and *FLZ17/18* were found to be responsive to prolonged starvation. These results suggest that majority of *FLZ* gene family members are responsive to sugar levels and cellular starvation. These results were further supported by GUS assay and 2DG treatment.

It is already well known that sugars transcriptionally regulate a large number of genes which belongs to diverse cellular processes. These sugar-regulated genes include genes involved in primary and secondary metabolism, hormone signaling and developmental processes such as phase transition, flowering, senescence etc. (Price et al., 2004; Li et al., 2006; Mishra et al., 2009; Xiong et al., 2013). Functional analysis of these genes deciphered molecular mechanisms by which sugars regulate different aspects of plant development. For example, the sugar-induced expression of nucelolin-1 in *A. thaliana* is important for ribosome synthesis (Kojima et al., 2007). Similarly, the glucose-TARGET OF RAPAMYCIN (TOR) signaling cascade regulates the transcription of many genes including cell cycle genes which is important in the establishment of early seedling growth (Xiong et al., 2013). From our analysis, it has been found that the cellular sugar and energy level is an important regulator of transcription of many *FLZ* genes. Similar functional analysis of individual *FLZ* genes will give more information on the molecular aspects of the sugar-mediated regulation of plant growth. Interestingly, in an earlier study, we have identified that most of the *FLZ* genes are differentially regulated during vegetative-to-reproductive phase transition (Jamsheer et al., 2015). The role of sugars on the developmental phase transition and flowering is already known. Sugars known to regulate these processes by controlling the activity of microRNAs and other well know regulators involved in the developmental phase transition and flowering (Seo et al., 2010; Wahl et al., 2013; Yang et al., 2013; Yu et al., 2013). The differential regulation of *FLZ* genes during vegetative-to-reproductive phase transition further supports the possible role of *FLZ* gene family in the sugar-mediated regulation of plant growth. The interaction of these proteins with kinase subunits of SnRK1 and regulatory subunit of TOR kinase also suggest their involvement of *FLZ* genes in the sugar-mediated regulation of plant growth (Arabidopsis Interactome Mapping Consortium, 2011; Nietzsche et al., 2014). Our transcriptional studies and earlier protein: protein interaction studies could suggest a link between sugars and *FLZ* gene family; however, more molecular studies are needed to establish this connection.

**FIGURE 7:**
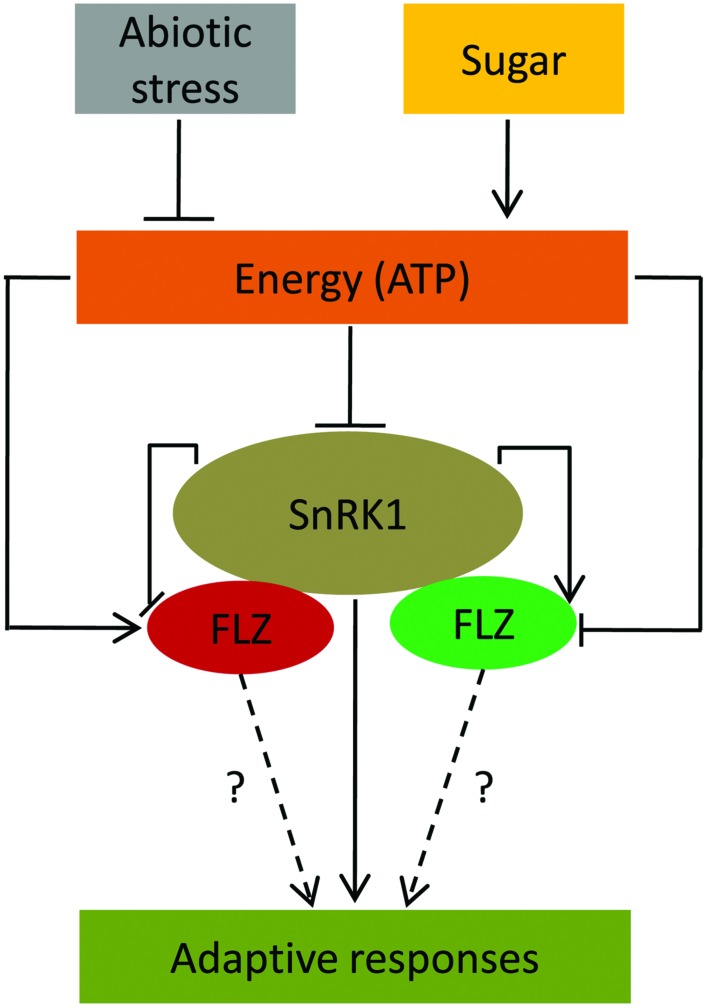
**A conceptual model suggesting *FLZ* genes as a factor involved in the regulation of adaptive response under energy fluctuations and abiotic stress.** Sugars, cellular energy level and abiotic stress regulate the expression of *FLZ* genes. Sugar-induced *FLZ* genes are shown in red color and sugar down-regulated genes are shown in green color. Stress-driven energy deficit activates SnRK1 which in turn activates the genes involved in the adaptive responses toward stresses (Baena-González et al., 2007; Cho et al., 2012; Confraria et al., 2013). *FLZ* genes are also involved in the stress mitigation (Hou et al., 2013). SnRK1 transcriptionally regulates *FLZ* genes. FLZ proteins physically interact with kinase subunits of SnRK1 (Arabidopsis Interactome Mapping Consortium, 2011; Nietzsche et al., 2014); however, the biological significance of these interactions is still unknown.

Transcriptional studies also identified that different abiotic stresses and ABA can regulate the expression of many *FLZ* genes. These results suggest the possible involvement of *FLZ* genes in mediating adaptive growth under abiotic stress conditions. There are already few reports which link *FLZ* genes in providing stress tolerance and involvement in ABA-directed processes. Over-expression of a wheat *FLZ* gene, *TaSRHP* in *A. thaliana* positively regulated the expression of many stress-related genes and enhanced resistance toward different abiotic stresses (Hou et al., 2013). The *FLZ9/MARD1* gene of *A. thaliana* was found to involved in ABA-mediated seed dormancy (He and Gan, 2004). Similarly, the interplay between sugars and stress signaling is well established. A large overlap between sugar- and stress-responsive genes is already reported by different groups suggesting the role of sugars and cellular energy in providing stress-tolerance (Price et al., 2004; Li et al., 2006). Disruption of many genes resulted in perturbation of both ABA- and sugar-mediated responses further suggest an overlap between glucose and stress signaling (Arenas-Huertero et al., 2000; Brocard et al., 2002). Our transcriptional studies firmly established that the *FLZ* genes are regulated by sugars, ABA, and abiotic stresses. Since sugar and cellular energy status is important in dealing stresses, this class of genes which are co-regulated by both sugars and stresses might be important in the regulation of adaptive responses toward stress and stress-derived energy depletion (**Figure 7**). The studies in this direction may open a novel molecular pathway by which sugars regulate adaptive growth during stress.

### The Sugar-Induced Expression of *FLZ* Genes is Dependent on HXK1-Dependent Signaling and Metabolism-Dependent Energy Signaling

Different glucose signaling pathways are implicated in the control of sugar-regulated gene expression in both distinct and overlapping manner (Miao et al., 2013; Xiong et al., 2013). In our study, we found that the sugar-induced expression of *FLZ* genes is regulated by two different sugar signaling pathways. Abolition of HXK1-dependent pathway reduced the expression of *FLZ* genes considerably after sugar treatment suggesting that HXK1-dependent signaling acts as a positive regulator of glucose-induced expression of *FLZ* genes. This pathway is independent of the kinase activity of HXK1 and decoupled from the sugar metabolism-dependent signaling (Moore et al., 2003). HXK1 form complex with H^+^-ATPase B1 and the 19S regulatory particle of proteasome subunit in the nucleus and sugar-regulated expression of many genes are reported to be dependent on this pathway (Cho et al., 2006; Lin et al., 2011; Miao et al., 2013). However, in case of sugar-induced *FLZ* genes, the abolition of the HXK1-dependent pathway could only partially hamper the transcript induction suggesting the involvement of other sugar signaling pathways.

The expression of *FLZ* genes was severely abolished when the cellular respiratory pathway was blocked independently at glycolysis and oxidative phosphorylation stages implying the role of metabolism-dependent energy signaling as the pivotal regulator of the sugar-induced *FLZ* gene expression. This observation is further supported by the contrasting response of *FLZ2* and *FLZ6* toward 2DG treatment. The metabolism dependent energy signaling pathway is implicated in controlling TOR and SnRK1 signaling (Nunes et al., 2013; Xiong et al., 2013). It is earlier reported that glucose-control of cell division, meristem activation, and the developmental transition is dependent on metabolism-dependent energy signaling pathway and independent of HXK1-dependent glucose signaling pathway. Understandably, all major hormone signaling pathways and the HXK1-dependent glucose signaling found to be downstream to the metabolism-dependent control of plant growth (Xiong et al., 2013). Our results suggest that induction of *FLZ* genes under sugar and energy rich conditions is controlled by metabolism-dependent energy signaling. The HXK1-dependent glucose signaling acts as a positive regulator of glucose-induced transcriptional up-regulation of *FLZ* genes. This regulation may be important for the fine tuning of *FLZ* gene function in response to sugar and energy signaling. Besides, the dual regulation of sugar-dependent transcriptional induction of *FLZ* genes by both HXK1-dependent glucose signaling and metabolism-dependent energy signaling suggest the possibility that *FLZ* genes might be acting as a cross-talking hub for different sugar signaling pathways established in plants.

### SnRK1 Regulation of *FLZ* Gene Transcription

The gene expression analysis in *KIN10* over-expression identified that SnRK1 regulates the transcription of *FLZ* genes. Interestingly, this regulation of SnRK1 on the transcription of *FLZ* genes found to have a connection with the sugar-response of these genes. The sugar-inducible *FLZ* genes were found to be repressed while the sugar-repressible genes were found to up-regulated in the *KIN10* over-expression line. The repression was found to be more severe in high sugar-inducible genes (Class I) compared to medium sugar-inducible gene *FLZ11* (Class II). Consistent with this hypothesis, it was found that *KIN10* positively regulates the expression of class III sugar-repressible genes *FLZ9* and *FLZ17*/*18*. These results provide a framework for the further studies regarding the role of *FLZ* gene in the regulation of stress responses, particularly during low-energy stress. This result suggests that SnRK1 is possibly working upstream to regulate the transcription of *FLZ* genes. It is already known that SnRK1 undertake a massive reprogramming of transcription during low-energy stress (Baena-González et al., 2007). Interestingly, the contrasting regulation of Class I and II genes and Class III genes in the *KIN10* over-expression line add more complexity to the situation. Molecular study of this sugar-response dependent regulation of *FLZ* genes by SnRK1 would reveal the further intricacies of adaptive growth under energy stress. The genetic studies using *FLZ* gene mutants and over-expression studies can tell whether these genes work downstream to SnRK1 in this pathway. Besides, it is known that both kinase subunits of SnRK1 physically interact with all FLZ proteins in *A. thaliana* (Arabidopsis Interactome Mapping Consortium, 2011; Nietzsche et al., 2014). Identification of biological significance of these interactions will be crucial in the elucidation of the molecular regulation of energy signaling-SnRK1 interaction. The FLZ proteins can be a downstream factor by acting as kinase substrate of SnRK1 or it is possible that this interaction regulate the kinase activity of SnRK1. Considering the regulation of sugar and energy level on SnRK1 activity and *FLZ* gene expression, both hypotheses deserve the merit for further studies. Low light and various abiotic stresses ultimately lead to energy deficit in the cell and the response of *FLZ* gene toward energy fluctuation and stress suggest that they are possibly involved in the regulation of adaptive responses which enable the plant to survive in these non-favorable conditions. More genetic, molecular and physiological studies are needed to decipher this pathway.

## Author Contributions

Conceived and designed the experiments: MJK and AL. Performed the experiments: MJK. Analyzed the data: MJK and AL. Contributed reagents/materials/analysis tools: MJK and AL. Wrote the paper: MJK and AL.

## Conflict of Interest Statement

The authors declare that the research was conducted in the absence of any commercial or financial relationships that could be construed as a potential conflict of interest.
