# Supplementary material for: Expression of Arabidopsis FCS-Like Zinc finger genes is differentially regulated by sugars, cellular energy level, and abiotic stress
Source: Front Plant Sci. 2015 Sep 24;6:746. doi: 10.3389/fpls.2015.00746 (PMC4585328; doi:10.3389/fpls.2015.00746)
Supplement: Supplementary file 1 [file Presentation_1.PPTX]

## Slide 1
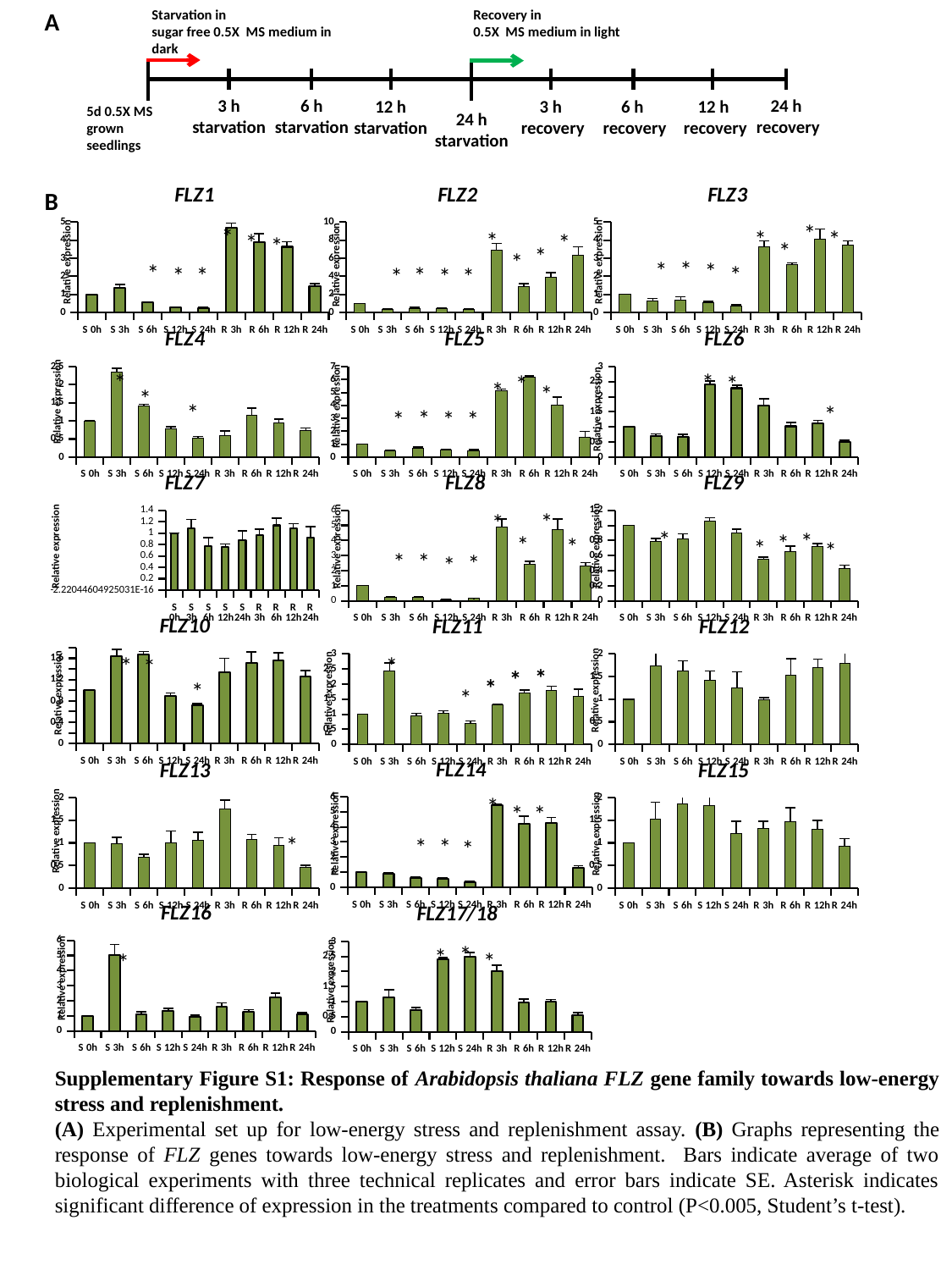

A
Starvation in
sugar free 0.5X MS medium in dark
Recovery in
0.5X MS medium in light
3 h starvation
6 h starvation
24 h
 recovery
12 h starvation
3 h
 recovery
6 h
 recovery
12 h
 recovery
5d 0.5X MS grown seedlings
24 h starvation
### Chart:
| Category | FLZ1 |
|---|---|
| S 0h | 1.0 |
| S 3h | 1.3424592278160241 |
| S 6h | 0.5496508334668072 |
| S 12h | 0.2667310058950423 |
| S 24h | 0.25932063991101073 |
| R 3h | 4.6938001893412995 |
| R 6h | 3.879000866214738 |
| R 12h | 3.6318605912331967 |
| R 24h | 1.4611228756419958 |
### Chart:
| Category | FLZ2 |
|---|---|
| S 0h | 1.0 |
| S 3h | 0.3300401186841313 |
| S 6h | 0.4466099325217315 |
| S 12h | 0.3838998171786711 |
| S 24h | 0.3382192028737064 |
| R 3h | 6.8576507191870455 |
| R 6h | 2.8518536571134567 |
| R 12h | 3.8815055974493426 |
| R 24h | 6.3077548316801755 |
### Chart:
| Category | FLZ3 |
|---|---|
| S 0h | 1.0 |
| S 3h | 0.6445616634601496 |
| S 6h | 0.6853120705269845 |
| S 12h | 0.5266700282802107 |
| S 24h | 0.3547293191209491 |
| R 3h | 3.6168659232019706 |
| R 6h | 2.634163520128183 |
| R 12h | 4.055237304698379 |
| R 24h | 3.7266631898191376 |B
*
*
*
*
*
*
*
*
*
*
*
*
*
*
Relative expression
Relative expression
*
Relative expression
*
*
*
*
*
*
*
### Chart:
| Category | FLZ4 |
|---|---|
| S 0h | 1.0 |
| S 3h | 2.3568544093676653 |
| S 6h | 1.4042780565008979 |
| S 12h | 0.7832204521292147 |
| S 24h | 0.5256512115102063 |
| R 3h | 0.5952772312147245 |
| R 6h | 1.1531745496185302 |
| R 12h | 0.9356505498984363 |
| R 24h | 0.7390006042856029 |
### Chart:
| Category | FLZ5 |
|---|---|
| S 0h | 1.0 |
| S 3h | 0.5080861272366075 |
| S 6h | 0.7054306239710012 |
| S 12h | 0.5561936134919176 |
| S 24h | 0.5006917493879112 |
| R 3h | 5.1141797869377745 |
| R 6h | 6.151194885883289 |
| R 12h | 4.03443936256962 |
| R 24h | 1.546919451936928 |
### Chart:
| Category | FLZ6 |
|---|---|
| S 0h | 1.0 |
| S 3h | 0.6888207933858218 |
| S 6h | 0.6796292746456953 |
| S 12h | 2.398631370121904 |
| S 24h | 2.284055137838317 |
| R 3h | 1.7191453954726126 |
| R 6h | 1.019500926711099 |
| R 12h | 1.1059030154472418 |
| R 24h | 0.49408146496763267 |*
*
*
*
*
*
*
Relative expression
*
*
Relative expression
*
*
*
*
Relative expression
### Chart:
| Category | FLZ7 |
|---|---|
| S 0h | 1.0 |
| S 3h | 1.090459430457094 |
| S 6h | 0.7733490425207438 |
| S 12h | 0.75820236420447 |
| S 24h | 0.8753908867263176 |
| R 3h | 0.9664212025980918 |
| R 6h | 1.136057481393421 |
| R 12h | 1.0779933049627435 |
| R 24h | 0.9216976927166517 |Relative expression
### Chart:
| Category | FLZ8 |
|---|---|
| S 0h | 1.0 |
| S 3h | 0.2298471581610574 |
| S 6h | 0.21134174858841126 |
| S 12h | 0.07506489251077647 |
| S 24h | 0.14266578299858668 |
| R 3h | 4.873458944474447 |
| R 6h | 2.4115919736927665 |
| R 12h | 4.708950916334727 |
| R 24h | 2.3108201629880467 |Relative expression
### Chart:
| Category | FLZ9 |
|---|---|
| S 0h | 1.0 |
| S 3h | 0.7863334917267786 |
| S 6h | 0.8264779310562426 |
| S 12h | 1.0516564821039518 |
| S 24h | 0.8971267430627186 |
| R 3h | 0.5507738376958149 |
| R 6h | 0.6505719575929894 |
| R 12h | 0.7195836924898745 |
| R 24h | 0.42872853197328264 |*
*
*
*
*
Relative expression
*
*
*
*
*
*
*
*
### Chart:
| Category | FLZ10 |
|---|---|
| S 0h | 1.0 |
| S 3h | 1.645438639176928 |
| S 6h | 1.6671642115092458 |
| S 12h | 0.8917163896769621 |
| S 24h | 0.7209431209079378 |
| R 3h | 1.3371028928609494 |
| R 6h | 1.5136420969186093 |
| R 12h | 1.5667754660644306 |
| R 24h | 1.265312902514204 |*
*
*
Relative expression
### Chart:
| Category | FLZ11 |
|---|---|
| S 0h | 1.0 |
| S 3h | 2.4235348185170107 |
| S 6h | 0.9422306432435414 |
| S 12h | 1.038259705823446 |
| S 24h | 0.6982923172322525 |
| R 3h | 1.3004844514521334 |
| R 6h | 1.6985980767685995 |
| R 12h | 1.7788999068224158 |
| R 24h | 1.5879381606051373 |*
*
*
*
*
Relative expression
### Chart:
| Category | FLZ12 |
|---|---|
| S 0h | 1.0 |
| S 3h | 1.7344683355935404 |
| S 6h | 1.6307802855790516 |
| S 12h | 1.4264726186328898 |
| S 24h | 1.24626541910626 |
| R 3h | 0.9947247690519595 |
| R 6h | 1.523455233016367 |
| R 12h | 1.7047982504964356 |
| R 24h | 1.7851338176290439 |Relative expression
### Chart:
| Category | FLZ14 |
|---|---|
| S 0h | 1.0 |
| S 3h | 0.8858264277389465 |
| S 6h | 0.6320537838385538 |
| S 12h | 0.5667341213887207 |
| S 24h | 0.34460025139290057 |
| R 3h | 5.461631110667478 |
| R 6h | 4.1931142561069725 |
| R 12h | 4.249152317668536 |
| R 24h | 1.2932107485245798 |*
*
*
Relative expression
*
*
*
### Chart:
| Category | FLZ13 |
|---|---|
| S 0h | 1.0 |
| S 3h | 0.9827960086218956 |
| S 6h | 0.6758927367792577 |
| S 12h | 0.9936707960443056 |
| S 24h | 1.0578825511988363 |
| R 3h | 1.7554168738688567 |
| R 6h | 1.0795239514650012 |
| R 12h | 0.9439092424073686 |
| R 24h | 0.44831596207180535 |Relative expression
*
### Chart:
| Category | FLZ15 |
|---|---|
| S 0h | 1.0 |
| S 3h | 1.5242998504724259 |
| S 6h | 1.856786370161868 |
| S 12h | 1.832953804682263 |
| S 24h | 1.2058495572498702 |
| R 3h | 1.3167020173769897 |
| R 6h | 1.4591549070522398 |
| R 12h | 1.308828029055448 |
| R 24h | 0.9150872262773655 |Relative expression
### Chart:
| Category | FLZ16 |
|---|---|
| S 0h | 1.0 |
| S 3h | 5.029376910270915 |
| S 6h | 1.1200456290690883 |
| S 12h | 1.3244287896004099 |
| S 24h | 0.9469524855896001 |
| R 3h | 1.5832391511388675 |
| R 6h | 1.264152135632237 |
| R 12h | 2.197665589975823 |
| R 24h | 1.0769848254441838 |*
Relative expression
### Chart:
| Category | FLZ17/18 |
|---|---|
| S 0h | 1.0 |
| S 3h | 1.129012091930783 |
| S 6h | 0.7106743255872532 |
| S 12h | 2.403214755343081 |
| S 24h | 2.486780271084079 |
| R 3h | 2.0241171090139916 |
| R 6h | 0.9728792058224947 |
| R 12h | 0.9923401020115746 |
| R 24h | 0.5544602309479213 |*
*
*
Relative expression
Supplementary Figure S1: Response of Arabidopsis thaliana FLZ gene family towards low-energy stress and replenishment.
(A) Experimental set up for low-energy stress and replenishment assay. (B) Graphs representing the response of FLZ genes towards low-energy stress and replenishment. Bars indicate average of two biological experiments with three technical replicates and error bars indicate SE. Asterisk indicates significant difference of expression in the treatments compared to control (P<0.005, Student’s t-test).

## Slide 2
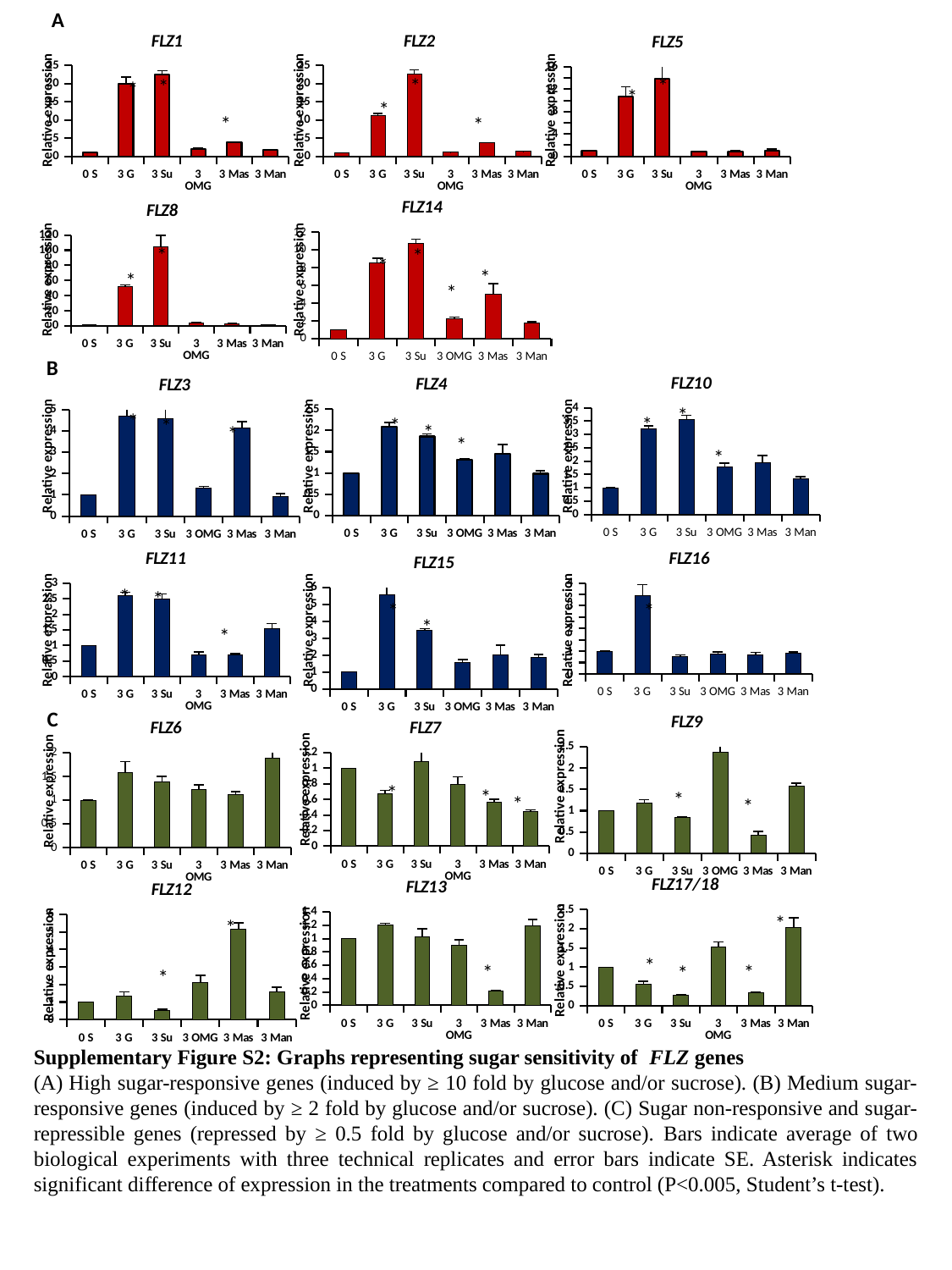

A
### Chart:
| Category | FLZ1 |
|---|---|
| 0 S | 1.0 |
| 3 G | 20.036448957027627 |
| 3 Su | 22.58123304123279 |
| 3 OMG | 1.9878414897259773 |
| 3 Mas | 3.9177590364575074 |
| 3 Man | 1.7261615802528738 |
### Chart:
| Category | FLZ2 |
|---|---|
| 0 S | 1.0 |
| 3 G | 11.323681769008656 |
| 3 Su | 22.625610121783083 |
| 3 OMG | 1.14373433406336 |
| 3 Mas | 3.705652801419079 |
| 3 Man | 1.3097399013735473 |
### Chart:
| Category | FLZ5 |
|---|---|
| 0 S | 1.0 |
| 3 G | 10.623654078846856 |
| 3 Su | 13.889530889060199 |
| 3 OMG | 0.8517797500193653 |
| 3 Mas | 0.834593931055346 |
| 3 Man | 1.0459189286752613 |*
*
*
*
*
*
Relative expression
Relative expression
Relative expression
*
*
### Chart:
| Category | FLZ14 |
|---|---|
| 0 S | 1.0 |
| 3 G | 8.519133106764329 |
| 3 Su | 10.705936738009967 |
| 3 OMG | 2.2370660230383677 |
| 3 Mas | 5.0474172272149245 |
| 3 Man | 1.7327062160730586 |
### Chart:
| Category | FLZ8 |
|---|---|
| 0 S | 1.0 |
| 3 G | 52.23073322454505 |
| 3 Su | 104.65781699234509 |
| 3 OMG | 3.5208742832881437 |
| 3 Mas | 2.725307454474115 |
| 3 Man | 1.3794300188598088 |*
*
*
*
*
Relative expression
Relative expression
*
B
### Chart:
| Category | FLZ10 |
|---|---|
| 0 S | 1.0 |
| 3 G | 3.214578688907389 |
| 3 Su | 3.5469376829277115 |
| 3 OMG | 1.7850146995259732 |
| 3 Mas | 1.9277852636012633 |
| 3 Man | 1.330859710048341 |
### Chart:
| Category | FLZ4 |
|---|---|
| 0 S | 1.0 |
| 3 G | 2.077198512605982 |
| 3 Su | 1.8572668427309178 |
| 3 OMG | 1.3032054149550023 |
| 3 Mas | 1.4553632309656133 |
| 3 Man | 0.9822288863356361 |
### Chart:
| Category | FLZ3 |
|---|---|
| 0 S | 1.0 |
| 3 G | 4.69601719019184 |
| 3 Su | 4.568159310252673 |
| 3 OMG | 1.2952543412585311 |
| 3 Mas | 4.135093618824057 |
| 3 Man | 0.9055329043920886 |*
*
*
*
*
*
*
*
*
Relative expression
Relative expression
Relative expression
### Chart:
| Category | FLZ11 |
|---|---|
| 0 S | 1.0 |
| 3 G | 2.5875672333505793 |
| 3 Su | 2.480641246402709 |
| 3 OMG | 0.7072565128798339 |
| 3 Mas | 0.696291292120313 |
| 3 Man | 1.5253992856752212 |
### Chart:
| Category | FLZ16 |
|---|---|
| 0 S | 1.0 |
| 3 G | 3.445787402469524 |
| 3 Su | 0.7777009829694183 |
| 3 OMG | 0.8701488672038984 |
| 3 Mas | 0.8321610676143346 |
| 3 Man | 0.8954383044277174 |
### Chart:
| Category | FLZ15 |
|---|---|
| 0 S | 1.0 |
| 3 G | 5.577439833333333 |
| 3 Su | 3.4484533333333327 |
| 3 OMG | 1.5500723333333364 |
| 3 Mas | 2.0285575000000002 |
| 3 Man | 1.877669833333333 |*
*
*
*
*
*
Relative expression
Relative expression
Relative expression
### Chart:
| Category | FLZ9 |
|---|---|
| 0 S | 1.0 |
| 3 G | 1.169761351288169 |
| 3 Su | 0.8380748685169319 |
| 3 OMG | 2.363087338717169 |
| 3 Mas | 0.4282145262423719 |
| 3 Man | 1.569188138568694 |C
### Chart:
| Category | FLZ6 |
|---|---|
| 0 S | 1.0 |
| 3 G | 1.57513693576132 |
| 3 Su | 1.3772438144809083 |
| 3 OMG | 1.227082320726466 |
| 3 Mas | 1.1169277715560137 |
| 3 Man | 1.8759985542370679 |
### Chart:
| Category | FLZ7 |
|---|---|
| 0 S | 1.0 |
| 3 G | 0.6678125385769511 |
| 3 Su | 1.083006500158526 |
| 3 OMG | 0.7930400530578151 |
| 3 Mas | 0.5671689155300726 |
| 3 Man | 0.44462044416744745 |*
Relative expression
Relative expression
*
Relative expression
*
*
*
### Chart:
| Category | FLZ17/18 |
|---|---|
| 0 S | 1.0 |
| 3 G | 0.5571597165397429 |
| 3 Su | 0.2641355965564959 |
| 3 OMG | 1.5159674417764535 |
| 3 Mas | 0.33645971506299216 |
| 3 Man | 2.0385490597308027 |
### Chart:
| Category | FLZ13 |
|---|---|
| 0 S | 1.0 |
| 3 G | 1.1981342474988574 |
| 3 Su | 1.0247902600989836 |
| 3 OMG | 0.8961698427598906 |
| 3 Mas | 0.21622911350327548 |
| 3 Man | 1.184576524116226 |
### Chart:
| Category | FLZ12 |
|---|---|
| 0 S | 1.0 |
| 3 G | 1.3242132623415401 |
| 3 Su | 0.5252764948564496 |
| 3 OMG | 2.1326597805782135 |
| 3 Mas | 5.13766380188174 |
| 3 Man | 1.5983696092190678 |*
*
*
Relative expression
Relative expression
Relative expression
*
*
*
*
Supplementary Figure S2: Graphs representing sugar sensitivity of FLZ genes
(A) High sugar-responsive genes (induced by ≥ 10 fold by glucose and/or sucrose). (B) Medium sugar-responsive genes (induced by ≥ 2 fold by glucose and/or sucrose). (C) Sugar non-responsive and sugar-repressible genes (repressed by ≥ 0.5 fold by glucose and/or sucrose). Bars indicate average of two biological experiments with three technical replicates and error bars indicate SE. Asterisk indicates significant difference of expression in the treatments compared to control (P<0.005, Student’s t-test).

## Slide 3
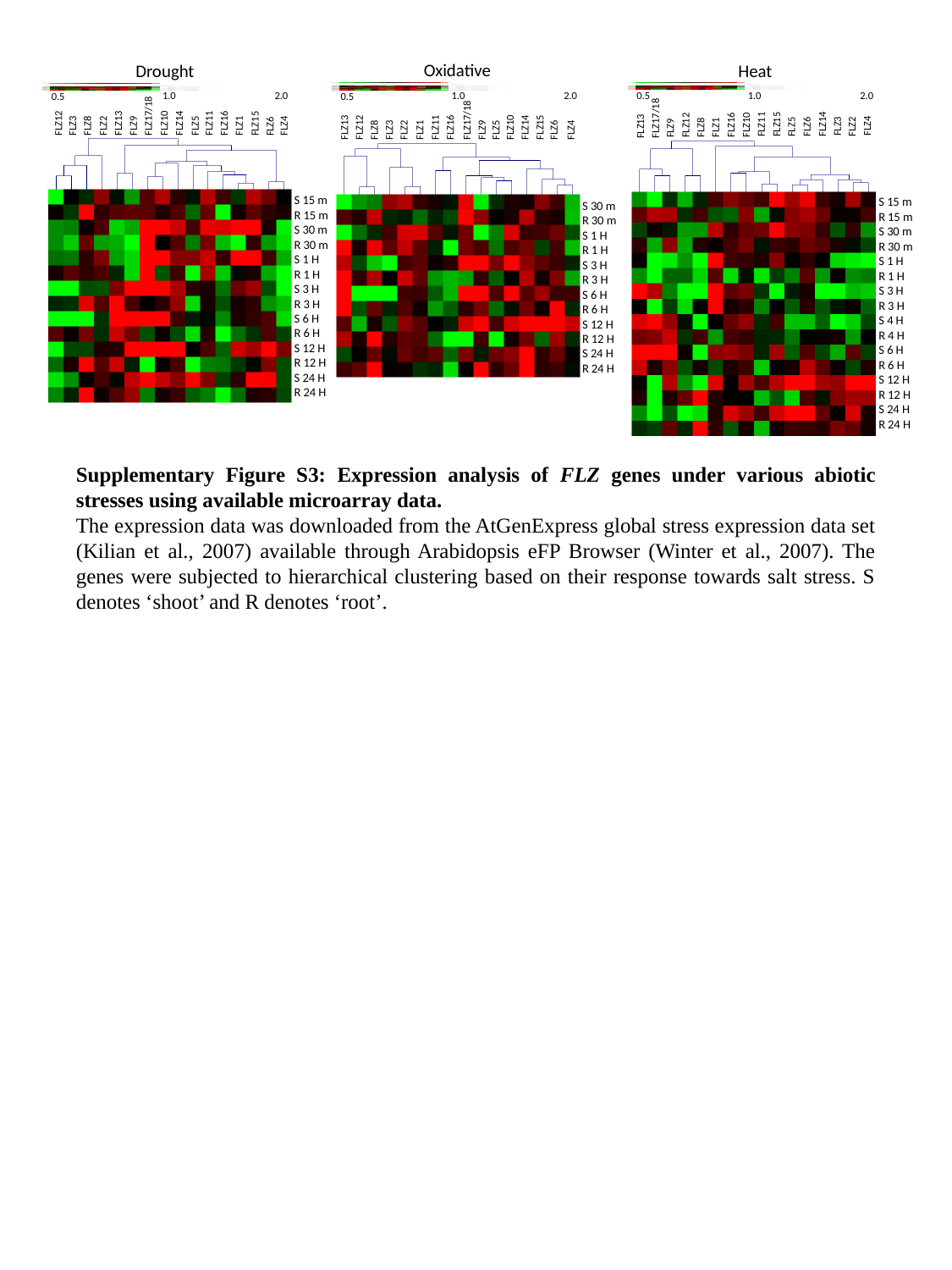

Oxidative
Drought
Heat
1.0
2.0
0.5
FLZ17/18
FLZ15
FLZ14
FLZ11
FLZ16
FLZ10
FLZ12
FLZ4
FLZ3
FLZ13
FLZ2
FLZ6
FLZ5
FLZ8
FLZ1
FLZ9
S 15 m
R 15 m
S 30 m
R 30 m
S 1 H
R 1 H
S 3 H
R 3 H
S 4 H
R 4 H
S 6 H
R 6 H
S 12 H
R 12 H
S 24 H
R 24 H
1.0
2.0
0.5
FLZ17/18
FLZ15
FLZ13
FLZ12
FLZ11
FLZ16
FLZ10
FLZ14
FLZ8
FLZ3
FLZ9
FLZ6
FLZ4
FLZ2
FLZ1
FLZ5
S 30 m
R 30 m
S 1 H
R 1 H
S 3 H
R 3 H
S 6 H
R 6 H
S 12 H
R 12 H
S 24 H
R 24 H
1.0
2.0
0.5
FLZ17/18
FLZ12
FLZ13
FLZ10
FLZ14
FLZ11
FLZ16
FLZ15
FLZ3
FLZ8
FLZ2
FLZ9
FLZ5
FLZ1
FLZ4
FLZ6
S 15 m
R 15 m
S 30 m
R 30 m
S 1 H
R 1 H
S 3 H
R 3 H
S 6 H
R 6 H
S 12 H
R 12 H
S 24 H
R 24 H
Supplementary Figure S3: Expression analysis of FLZ genes under various abiotic stresses using available microarray data.
The expression data was downloaded from the AtGenExpress global stress expression data set (Kilian et al., 2007) available through Arabidopsis eFP Browser (Winter et al., 2007). The genes were subjected to hierarchical clustering based on their response towards salt stress. S denotes ‘shoot’ and R denotes ‘root’.

## Slide 4
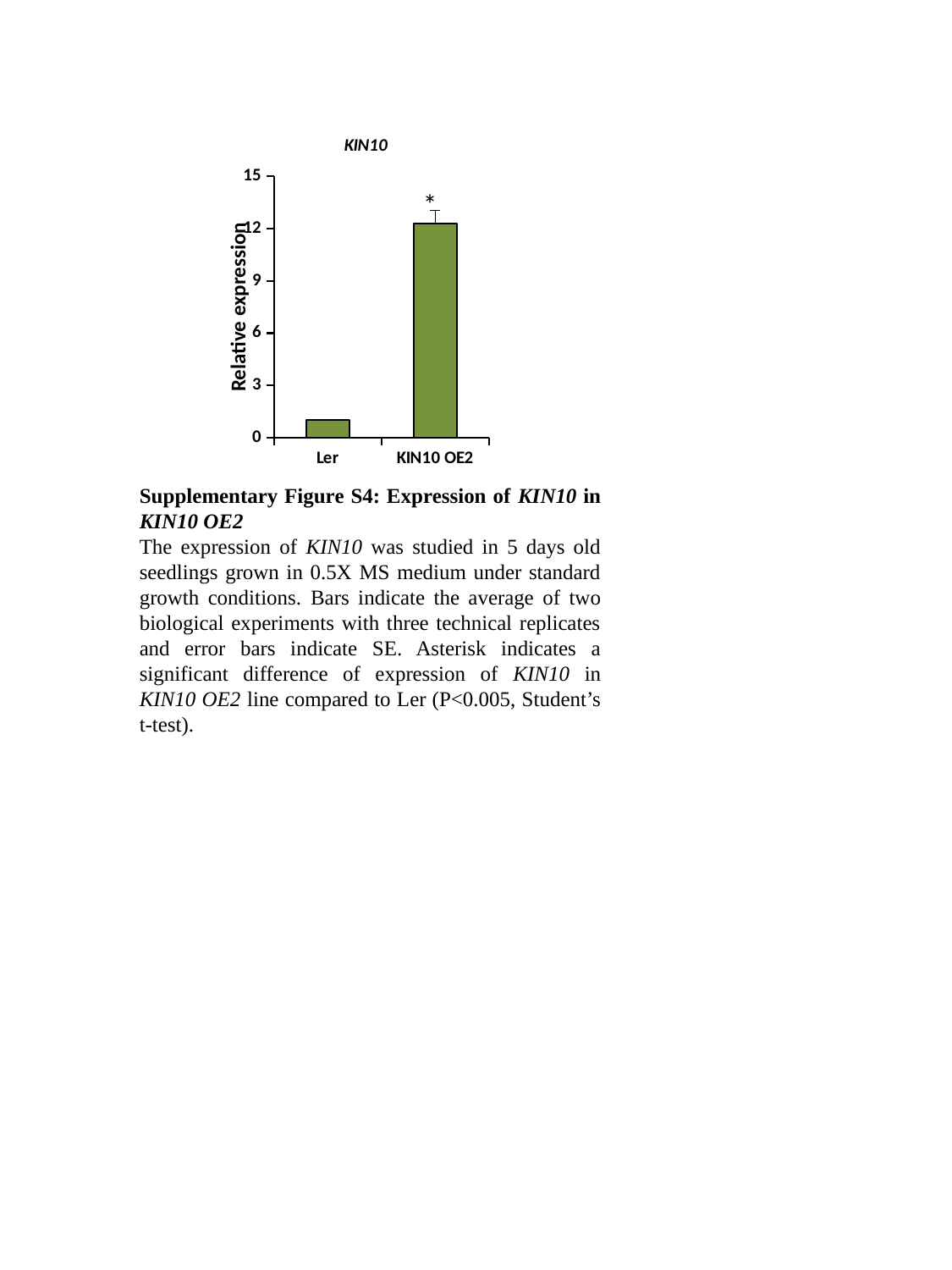

### Chart:
| Category | KIN10 |
|---|---|
| Ler | 1.0 |
| KIN10 OE2 | 12.301939687468504 |*
Relative expression
Supplementary Figure S4: Expression of KIN10 in KIN10 OE2
The expression of KIN10 was studied in 5 days old seedlings grown in 0.5X MS medium under standard growth conditions. Bars indicate the average of two biological experiments with three technical replicates and error bars indicate SE. Asterisk indicates a significant difference of expression of KIN10 in KIN10 OE2 line compared to Ler (P<0.005, Student’s t-test).
