# Supplementary material for: Expression of Arabidopsis FCS-Like Zinc finger genes is differentially regulated by sugars, cellular energy level, and abiotic stress
Source: Front Plant Sci. 2015 Sep 24;6:746. doi: 10.3389/fpls.2015.00746 (PMC4585328; doi:10.3389/fpls.2015.00746)
Supplement: Supplementary file 2 [file Table_1.DOCX]

| **Supplementary Table S1:** Primers used in this study | |
| --- | --- |
| Primers used for cloning | |
| Promoter_FLZ1 F | TTTTATGGGATTGCAATTGTATT |
| Promoter_FLZ1 R | GATGTGAAGAAAGTGTGAAGTGG |
| Promoter_FLZ6 F | CCATGGGTTTGGTTACTGTAGAATAATATA |
| Promoter_FLZ6 R | CTGCAGAGTGAGGAAGGAGAAGAG |
| Promoter_FLZ8 F | CCATGGCTTACTTAATAACAATTATAAAC |
| Promoter_FLZ8 R | CTGCAGAATAATAATACTTATATGAATCAC |
| Primers used for qRT-PCR | |
| FLZ1 F | GAAGCTGGGTTTTCTGGTAACAA |
| FLZ1 R | TAAGAGAAACGGCTTGAAGAAACG |
| FLZ2 F | GAAGACGATGGTTTTGTTTCTTTATCT |
| FLZ2 R | TCTGAGGATAGTTGTAACAAGAAGGACTT |
| FLZ3 F | GTATTACTCTGGTTTTTTGGGTTGTG |
| FLZ3 R | TCCGGCAAAGAGAACACGAT |
| FLZ4 F | CCCACGATCTATGACGCATCT |
| FLZ4 R | TGTGAGGGTGTTGATGAAAGGT |
| FLZ5 F | CGGAACCACCACAGGAGAGA |
| FLZ5 R | TCACAGTCAATAGTGCTCAATGCTT |
| FLZ6 F | TGTAACAGCAGCCGTTGATCA |
| FLZ6 R | ACCATGGATAAGAGCCGTTGA |
| FLZ7 F | CGCAGCGTTTTGTAGCATAGAAT |
| FLZ7 R | GACGCGGTTTCTTGTTTTCC |
| FLZ8 F | CCAGAATCATCTCCGGCTATTTC |
| FLZ8 R | CGCATGTGTAATCCTCCGATAAC |
| FLZ9 F | TCGAGCCAACTATCCCTAATCC |
| FLZ9 R | GAGATCGGCTAGGCCAAAGA |
| FLZ10 F | TCCGTCGATGCTGCTTACTG |
| FLZ10 R | GACAAGCATTGTTGTTCACACTGA |
| FLZ11 F | TGTGGCCCTGCTGCTAAAG |
| FLZ11 R | CCGATCATCGCCAAGAGAA |
| FLZ12 F | CCCGTCGCCTGGAAGTT |
| FLZ12 R | AAGCGCAGCGACGATACCTA |
| FLZ13 F | CGAAGCGGTTCGGTTCTG |
| FLZ13 R | AGTTTCCTCCAACGCAGCAA |
| FLZ14 F | AAATGATGAAAGGAAAGAGAGATGTAGA |
| FLZ14 R | TTTGGCCGGCGGTGTA |
| FLZ15 F | TGCGCCGTCGCGTTA |
| FLZ15 R | AGCGAAACCGCCTGCTT |
| FLZ16 F | GGAAGAACATCCAAAGATGAGG |
| FLZ16 R | GATGATGGTGATGATGAGATCCTA |
| FLZ17/18 F | GAAGATGATGTTGGCTTCGTACAG |
| FLZ17/18 R | ACAAATTCCGAGACTCGCTTTT |
| KIN10 F | GCGCAGATGGTATGCTCAGTAA |
| KIN10 R | TGCTGGACTCGTCTCCAAAGT |
